# Retracted: Hyperglycemia Induces Toll-Like Receptor-2 and -4 Expression and Activity in Human Microvascular Retinal Endothelial Cells: Implications for Diabetic Retinopathy

**DOI:** 10.1155/2020/5071954

**Published:** 2020-10-03

**Authors:** Journal of Diabetes Research

*Journal of Diabetes Research* has retracted the article titled “Hyperglycemia Induces Toll-Like Receptor-2 and -4 Expression and Activity in Human Microvascular Retinal Endothelial Cells: Implications for Diabetic Retinopathy” [1] due to concerns identified with Figures 1 and 2.

Following the publication of an erratum to correct an image duplication in Figure 2 [2], concerns have been identified in the original publication, and in the revised figure provided by the authors. Our concerns are as follows:

Figure 1(a)

- GADPH bands C and 15 are the same as bands 25 and M (horizontally flipped).
- In the TLR4 bands, there is an undeclared gel splice between the C and 15 bands

Figure 1(b)

- There is an undeclared gel splice between the 25 and M lanes of TLR4 which is not consistent with the loading control

Figure 2(c)

- There is an undeclared gel splice between the C and 15 lanes of TRIF and IRF3
- There is an undeclared gel splice between the 25 and M lanes of TRIF and IRF3
- These gel splices are not consistent with the loading control

With the agreement with the Chief Editor, this article is therefore being retracted due to the above concerns.
